# Functional Network Profiles in ARSACS Disclosed by Aptamer-Based Proteomic Technology

**DOI:** 10.3389/fneur.2020.603774

**Published:** 2021-01-27

**Authors:** Federica Morani, Stefano Doccini, Giovanna Chiorino, Fabiana Fattori, Daniele Galatolo, Elisa Sciarrillo, Federica Gemignani, Stephan Züchner, Enrico Silvio Bertini, Filippo Maria Santorelli

**Affiliations:** ^1^Molecular Medicine for Neurodegenerative and Neuromuscular Diseases Unit, IRCCS Stella Maris Foundation, Pisa, Italy; ^2^Department of Biology, University of Pisa, Pisa, Italy; ^3^Laboratorio di Genomica, Fondazione Edo ed Elvo Tempia, Biella, Italy; ^4^Unit of Muscular and Neurodegenerative Disorders, Department of Neurosciences, Bambino Gesù Children's Hospital, Rome, Italy; ^5^Department of Human Genetics, Hussman Institute for Human Genomics, University of Miami, Miami, FL, United States

**Keywords:** ARSACS, SomaLogic technology, sacsin, proteomic analysis, synaptogenesis, neuroinflammation, engulfment of cells

## Abstract

Although the genetic basis of autosomal recessive spastic ataxia of Charlevoix-Saguenay (ARSACS) has been uncovered, our poor understanding of disease mechanisms requires new light on functional pathways and modifying factors to improve early diagnostic strategies and offer alternative treatment options in a rare condition with no cure. Investigation of the pathologic state combining disease models and quantitative omic approach might improve biomarkers discovery with possible implications in patients' diagnoses. In this study, we analyzed proteomics data obtained using the SomaLogic technology, comparing cell lysates from ARSACS patients and from a *SACS* KO SH-SY5Y neuroblastoma cell model. Single-stranded deoxyoligonucleotides, selected *in vitro* from large random libraries, bound and quantified molecular targets related to the neuroinflammation signaling pathway and to neuronal development. Changes in protein levels were further analyzed by bioinformatics and network approaches to identify biomarkers of ARSACS and functional pathways impaired in the disease. We identified novel significantly dysregulated biological processes related to neuroinflammation, synaptogenesis, and engulfment of cells in patients and in KO cells compared with controls. Among the differential expressed proteins found in this work, we identified several proteins encoded by genes already known to be mutated in other forms of neurodegeneration. This finding suggests that common dysfunctional networks could be therapeutic targets for future investigations.

## Introduction

Autosomal recessive spastic ataxia of Charlevoix-Saguenay (ARSACS) is a rare disease caused by mutations in *SACS*, the gene encoding sacsin (1). ARSACS is characterized by early-onset spasticity in the lower limbs, axonal-demyelinating sensorimotor peripheral neuropathy, and cerebellar ataxia (2–4). The disease is usually diagnosed in early childhood, at the time of acquisition of independent walking, but late-onset, adult forms have occasionally been reported (5).

The availability of disease biomarkers in peripheral tissues, assessable using simple, non-invasive techniques, would help to shed light on the impaired biological pathways underlying the disease and could be important for early diagnosis.

Proteomics, the massive analysis of proteins, is now feasible using various technologies, such as mass spectrometry, gel-based techniques, antibody-based arrays, and recently developed aptamer-based technologies (6–8). Despite these technological advances, the extraction of knowledge from generated data is still challenging and requires significant bioinformatics expertise. To address this challenge, network analysis could enable the integration of highly descriptive disease biomarkers with existing knowledge and could potentially provide additional insights into the biological processes involved (9, 10).

In this study, capitalizing on our early work investigating the mRNA signature in neuronal models of ARSACS (11), we used a targeted proteomics assay as the starting point of an analysis aimed at identifying the neuronal pathways that are impaired in this disease. We used SOMAscan, a new aptamer-based proteomics assay that, by means of innovative and specific SOMAmer-based DNA signals, can quantitatively detect proteins in biological samples up to femtomolar concentration (7, 8). After assaying 1,300 SOMAmer-associated putative biomarkers in cultured skin fibroblasts from healthy controls and ARSACS patients, and in wild-type (WT) and sacsin knocked-out (KO) SH-SY5Y cells, we used functional network annotation to identify new major dysregulated biological processes in our models, namely neuroinflammation, synaptogenesis, and engulfment of cells. In particular, a short-term treatment with FCCP was used in SH-SY5Y cells to mimic the pathological phenotype related to defects in mitochondrial function and dynamics, as previously demonstrated by us and other (11, 12).

Bioinformatic analyses of these early proteomic signatures in ARSACS appear to replicate data previously observed in *Sacs*^−/−^ mouse models (12–14) and suggest a connection with mechanisms leading to neurodegeneration in spastic ataxia.

Identification of the underlying mechanisms in ARSACS might not only improve our biological knowledge of the way sacsin operates in disease conditions but could also offer new targets for a timelier diagnosis and open up future pharmacological opportunities for patients.

## Materials and Methods

### Collection and Processing of the Samples

In a previous work, we generated and characterized the SH-SY5Y neuroblastoma cell line and derived KO and WT clones (11). In brief, using CRISPR/Cas9 gene editing, we produced clones harboring a loss-of function mutation in *SACS*, and in RNAseq experiments compared transcriptomic profiles with WT clones. Cells were grown at 37°C with 5% CO_2_ in MEM/F12 1:1 with 10% fetal bovine serum (FBS), 2 mM l-glutamine, and 1% penicillin/streptomycin. Cells were treated with 20 μM carbonyl cyanide-4-(trifluoromethoxy) phenylhydrazone (FCCP) for 2 h to induce mitochondrial damage and fragmentation. Human fibroblasts were collected from diagnostic punch-skin biopsies using standard procedures and with signed informed consent. Primary fibroblast cell lines from three ARSACS patients presenting severe disease status and carrying different mutations in *SACS* (Supplementary Table 1) and from three healthy subjects were grown at 37°C with 5% CO_2_ in Dulbecco's modified Eagle's medium, containing 10% FBS, 4.5 g/L glucose and 1% antibiotics/antimycotics. All chemicals came from Sigma-Aldrich (St. Louis, MO). All samples (fibroblasts and cell models) were washed twice with PBS 1X and then homogenized in M-PER™ Mammalian Protein Extraction Reagent (Thermo Scientific, Rodano (MI), Italy) containing inhibitors of proteases (Roche Diagnostics, Monza (MB), Italy), following the manufacturer's standard protocol. About 200 μg/mL of cell proteins, measured by BCA assay (Invitrogen-Thermo Fisher Scientific, Waltham, MA) were placed in a final volume of 75 μL of PBS, ready for SOMAscan assay.

### SomaLogic Proteomic Technology and Analysis

Protein expressions were quantified using the validated SOMAscan technology (SomaLogic, Inc., Boulder, CO) (https://somalogic.com/technology/publications). A SOMAmer-protein binding step was followed by partitioning and wash steps that convert relative protein concentrations into measurable nucleic acid signals; these signals were quantified using hybridization to custom DNA microarrays, as already reported (7, 15). The readout in relative fluorescent units was directly proportional to the amount of target protein in the initial sample, as informed by a standard curve generated for each protein-SOMAmer pair (16).

Sample data were first normalized to remove hybridization variation within a run; this step was followed by normalization and calibration across all samples to remove other biases both within and between runs. Signal intensities in each group (SH-SY5Y cells and cultured skin fibroblasts) were normalized separately and processed at the Genomic Laboratory, Fondazione Edo ed Elvo Tempia, Biella, Italy.

### Statistical Analysis

The significant analysis of microarray method was used based on a set of sample permutations and performed as described (17). The *siggenes* R package was used to identify lists of differentially expressed proteins in cultured skin fibroblasts from ARSACS patients vs. healthy controls and KO vs. WT neuronal-like cell lines, treated or untreated. Lists of statistically significant positive (upregulated) and negative (downregulated) differentially expressed proteins (DEPs) were retrieved by setting the fold-change (FC) threshold higher than 1.25 and the *p*-value threshold lower than 0.01.

### Functional Network Analyses

Sets of DEPs in each sample were categorized using the Ingenuity Pathway Analysis (IPA) molecular suite (IPA Spring Release, Mar 2020, QIAGEN, Hilden, Germany, https://www.qiagenbioinformatics.com/products/ingenuity-pathway-analysis) to interpret datasets in the context of biological processes, pathways, and molecular networks. Expression analysis was based on protein FC, to calculate directionality (*z*-score) in the analysis, whereas the Ingenuity Knowledge Base reference set was used for *p*-value calculation.

We carried out a *core analysis* and *comparison analysis* to correlate DEPs with a given biological function, estimating its predicted activation or inhibition. Using a calculated *z*-score, to compare the independent *core analyses* of SH-SY5Y cells and skin fibroblasts, we selected the most meaningful functional annotations common to each group.

To link DEPs belonging to the different ARSACS experimental models, we used the IPA *Pathway Explorer* tool (18), setting only direct interactions involved in neurological diseases. To discover whether significant DEPs found in ARSACS might have a potential causative or modifier role in other forms of spastic ataxia or neurodegenerative conditions, we explored the genomic collection of the Prepare-Ataxia research network (https://www.prepare-ataxia.com) and queried the GENESIS 2.0 platform (https://www.tgp-foundation.org/genesis-log-in) (19), an affordable genome-scale analysis and data management solution for medical research containing genomic data of over 9,000 individuals with rare neurological diseases. Using a filtering scheme already reported in Lieto et al. (20), we performed matchmaking for DEPs if variants met the following criteria: (1) non-synonymous and loss-of-function mutations; (2) Combined Annotation Dependent Depletion score (https://cadd.gs.washington.edu/) >25 for missense mutations (21); (3) adaptive boosting and random forest scores > 0.6 as an index of splicing impact (larger scores indicate more likely splice altering) (22); (4) minor allele frequency (MAF) <0.01 or absent in public databases including gnomAD (https://gnomad.broadinstitute.org/); and (5) homozygous count <2 in either gnomAD or GENESIS 2.0.

## Results

### Comparative Analysis and Bioinformatics Categorization of Target Protein Expression

We ran three technical replicates per sample on the multiplexed proteomic platform using the SOMAScan Assay technology to investigate a targeted set of 1,300 proteins and compare their expression in skin fibroblasts from ARSACS patients and healthy subjects and in neuroblastoma WT and KO cell lines, either untreated or treated with FCCP, the uncoupler of mitochondrial oxidation and phosphorylation leading to impaired mitochondrial fragmentation (11).

By means of this strategy, we quantified differentially expressed proteins filtered by a FC threshold (>25% both in upregulation and downregulation) and a strict statistical significance threshold (*p*-value < 0.01) for all comparisons. Supplementary Table 2 lists the DEPs we had prioritized. In ARSACS patients, we identified a total of 25 DEPs (21 up- and four downregulated), whereas we found 93 DEPs (42 up- and 51 downregulated) and 76 DEPs (11 up- and 65 downregulated) in untreated and FCCP-treated KO cells, respectively. The full lists of DEPs could feed the IPA qualitative molecular suite in order to define a more precise network, core and comparative analyses.

Using an IPA-Core Analysis workflow, we interpreted datasets in the context of biological processes, pathways, and molecular networks and attempted to understand our results in the context of biological systems using the Ingenuity Knowledge Base as the background reference set.

**Figure 1:**
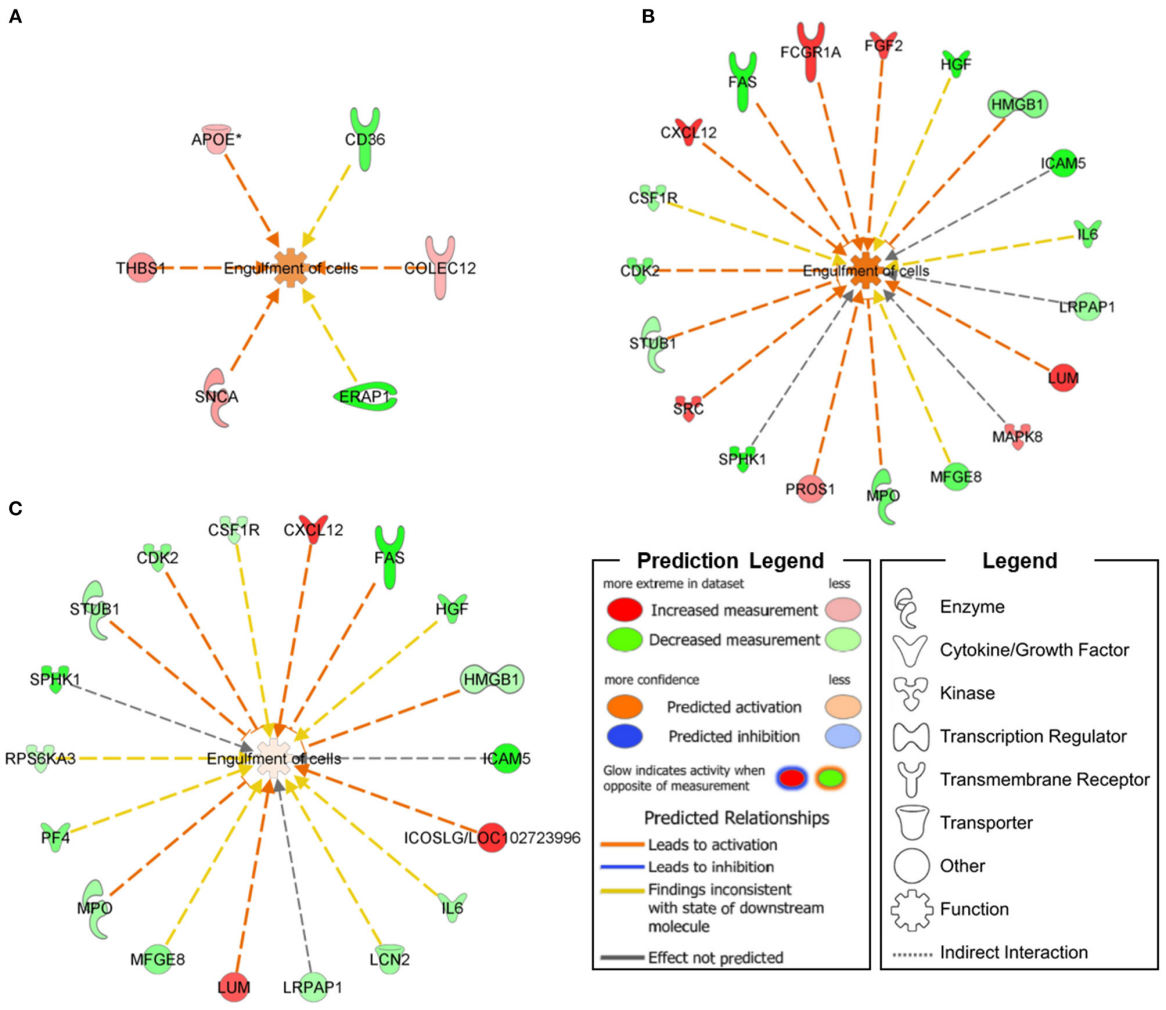
Functional networks connecting *Engulfment of Cells* annotations with DEPs specifically expressed in ARSACS patients **(A)** and *SACS* KO model under basal conditions **(B)** or after short-term FCCP treatment **(C)**. An upregulation of *Engulfment of Cells* was transversely observed in the three analyzed models.

**Figure 2:**
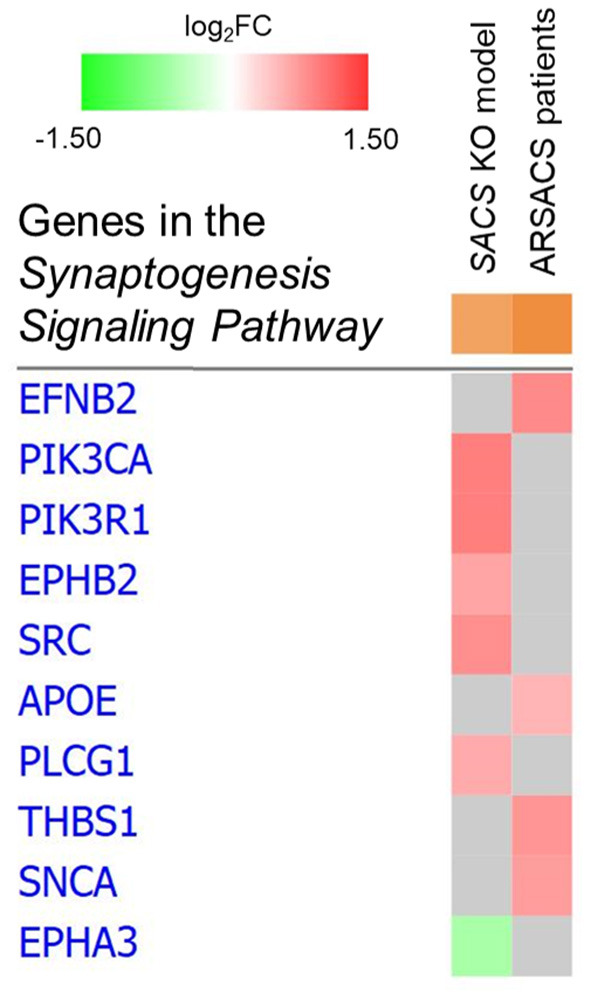
Heat map of *Synaptogenesis Signaling Pathway* in *SACS* KO model and ARSACS patients. No common dysregulated proteins were identified between models.

In skin fibroblasts, we defined two top-level functional networks related to *Engulfment of Cells* (–log_10_ *p*-value = 3.79; molecules = 6, Figure 1A) and *Synaptogenesis Signaling Pathway* (–log_10_ *p*-value = 3.39; molecules = 4, Figure 2) in order to explore the molecular suite for the *Disease & Function* and *Canonical Pathway* categories.

In SH-SY5Y cells, we found involvement of the *Engulfment of Cells* (–log_10_ *p*-value = 9.82; molecules = 19, Figure 1B) and *Neuronal Cell Death* (–log_10_ *p*-value = 17.42; molecules = 30) functional networks in the *Disease & Function* category, together with impairment of two *Canonical Pathways*—*Neuroinflammation Signaling Pathway* (–log_10_ *p*-value = 7.38; molecules = 11) and *Synaptogenesis Signaling Pathway* (–log_10_ *p*-value = 2.75; molecules = 6, Figure 2).

Following further scrutiny of the main functional annotations in KO cells treated with the uncoupling FCCP, we also noticed an overall reduction of the dysregulation level of proteins involved in *Engulfment of Cells* (–log_10_ *p*-value = 9.82; molecules = 19, Figure 1C) and *Neuroinflammation Signaling Pathway* (–log_10_ *p*-value = 7.74; molecules = 11).

*Synaptogenesis Signaling Pathway* DEPs were found to be distributed along the presynaptic (SNCA, EFNB2) as well as the post-synaptic terminal (PIK3CA, PIK3R1, PLCG1, EPHA3, EPHB2) and the synaptic cleft (THBS1, APOE). We also identified four proteins (ICAM5, SPHK1, STUB1, and SNCA) with a neuronal role in the *Engulfment of Cells* functional network, and two cytokines (IL-6 and CXCL12) involved in neuromodulation (23–25); SNCA was also found to be implicated in cerebellar inflammation and oxidative stress (26) as part of the *Neuroinflammation Signaling Pathway*.

### Integrated Proteomics Analysis Between Models

A single protein, NAGK (ID: Q9UJ70), was found to be downregulated both in cell models and in skin cells from patients. NAGK is implicated in sugar metabolism and was recently found to be highly expressed in neuronal dendrites; it appears important in axonal growth of developing neurons (27). However, the link with the abovementioned functional networks is unclear.

**Figure 3:**
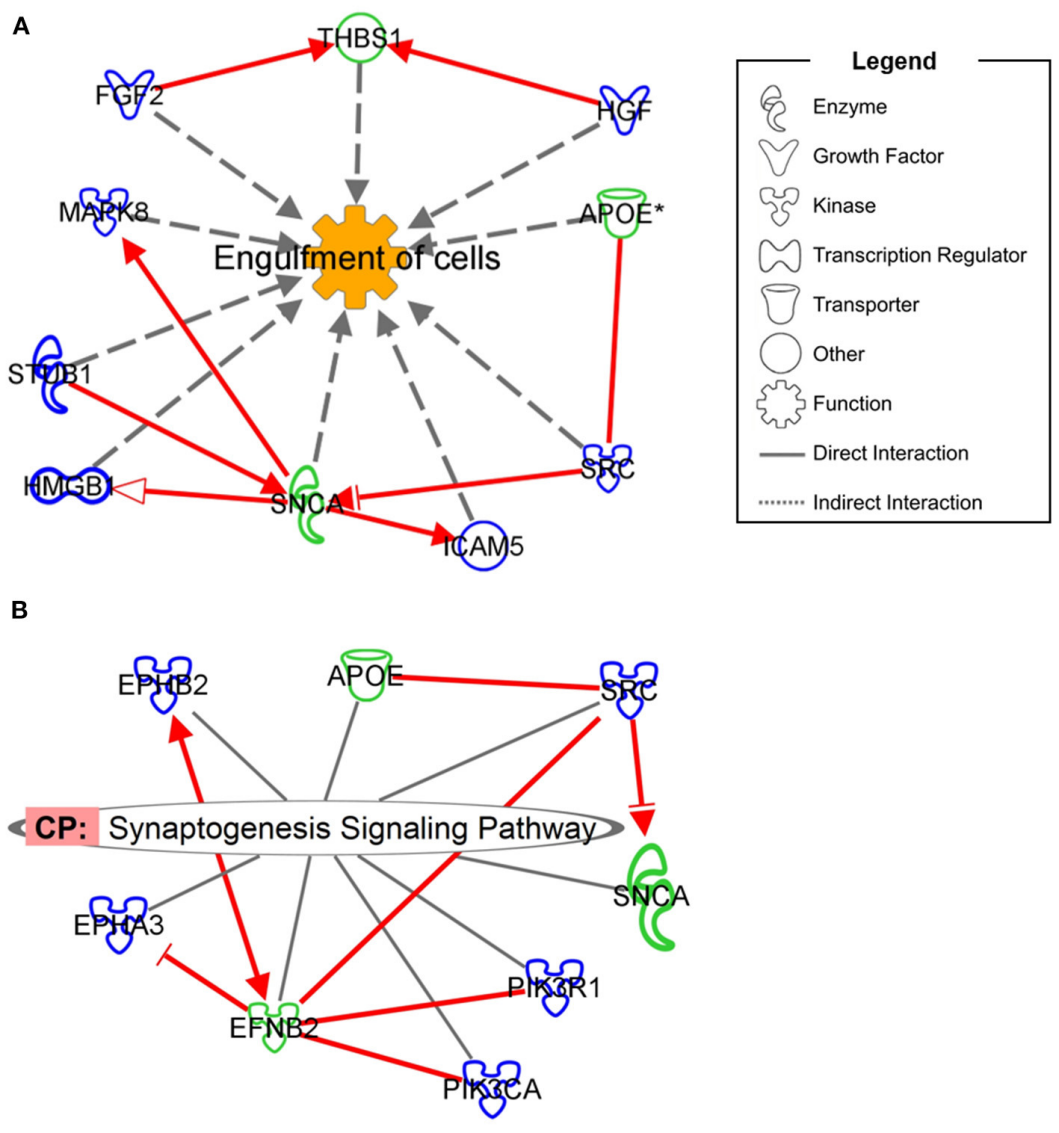
Interaction network showing the mutual involvement of DEPs from *SACS* KO model and ARSACS patients in *Engulfment of Cells* **(A)** and *Synaptogenesis Signaling Pathway* **(B)**. DEPs in patients are shown with green outlining while DEPs in the neuroblastoma model are shown with blue outlining. Only direct interaction between DEPs belonging to different models and involved in neurological disease was considered eligible (red arrows).

Although no simple biomarker emerged from our analyses, integration of proteomics information from ARSACS patients and from a neuronal-like cell model revealed a specific interaction for neurological diseases. As shown in Figure 3, we found that seven DEPs in the SH-SY5Y model could interact with ARSACS cell lines in the *Engulfment of Cells* pathway (Figure 3A), and that five DEPs showed similar interactions in the *Synaptogenesis Signaling Pathway* (Figure 3B). These findings indicate that the dysregulated DEPS are closely linked in functional pathways contributing to disease mechanisms. It is noteworthy that among the interconnected DEPs, one protein (SNCA) is known to be expressed in Purkinje neurons (26), the main target of sacsin dysfunction, whereas another (EFNB2) is related to neurodevelopment (28), a critical aspect of ARSACS (29).

### Proteomic Crosstalk Between Neurological Diseases

**Figure 4:**
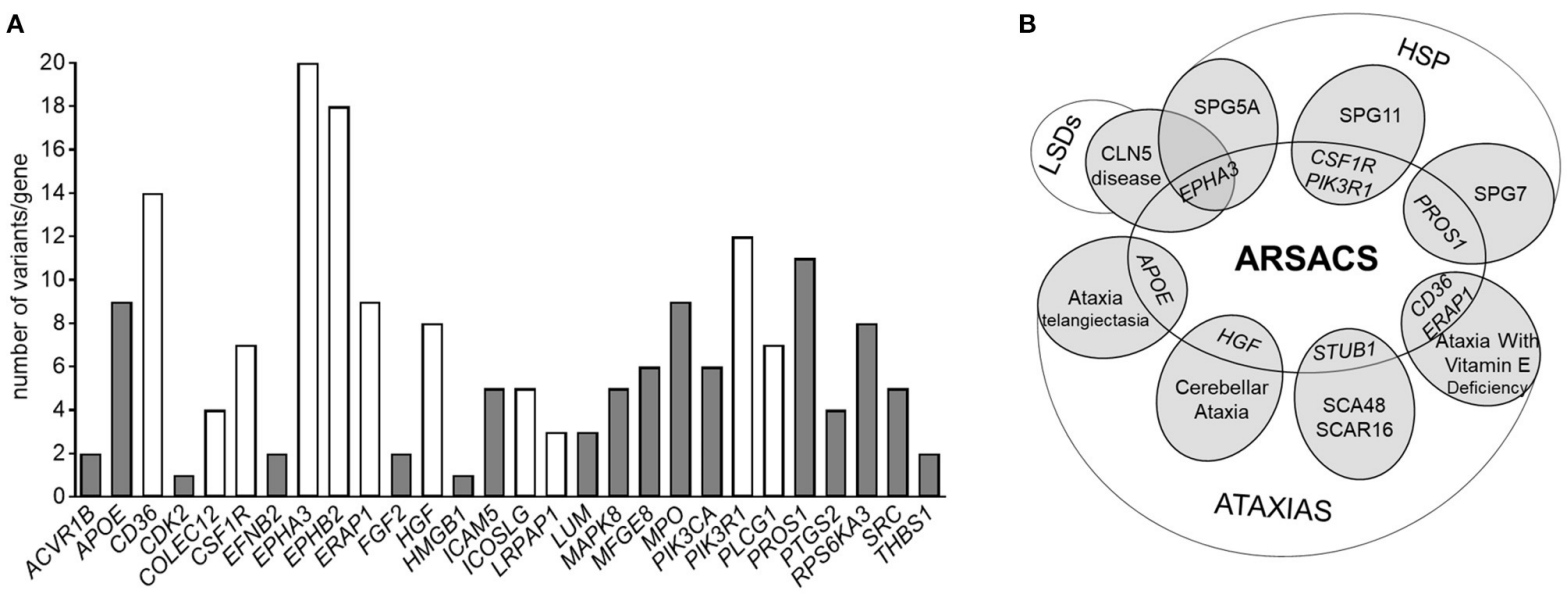
**(A)** Histogram showing the number of pathogenic variants in the genes encoding DEPs present in our datasets, associated with a phenotype of hereditary ataxia or hereditary spastic paraplegia. Variants reported as common to more than one neurological phenotype are reported in white. **(B)** Venn diagram showing a speculative overlap of genes encoding some of the identified DEPs in our dataset and rare and deleterious variants underlying other neurodegenerative disorders obtained by Genesis 2.0. Variants in STUB1 were not obtained by Genesis queries, but are included here because of its pathogenic significance.

When interrogating the GENESIS genomic analysis platform to disclose possible variants in genes encoding a subset of 38 selected DEPs identified in our study, we prioritized 188 putatively pathogenic variants found in 28 genes in 140 cases (Supplementary Table 3_A). Interestingly, the fact that 22 of these variants are found in patients with amyotrophic lateral sclerosis or Charcot-Marie-Tooth disease or ataxia-spasticity spectrum disorders (Figure 4A and Supplementary Table 3) suggests a particular involvement of those genes (and their products) in the pathways underlying neurodegeneration.

Ten variants (in eight genes) occurred in patients with an already genetically characterized form of spastic ataxia (SPAX) (Supplementary Table 3_B), giving us the impression that they might be potential modifier factors in ARSACS (Figure 4B).

It is worth noting that 36 individuals with undiagnosed ataxia harbored at least two putative pathogenetic mutations in DEP-encoding genes and that nine of these share the same combination of multiple mutated genes (*EPHA3/EPHB2, EPHB2/SRC, EPHA3/MPO, APOE/PIK3CA*), encoding proteins that are mostly part of the *Synaptogenesis Signaling Pathway*.

## Discussion

The SOMAscan platform, allowing the proteomic profiling of numerous samples, makes it possible to discover biomarkers potentially useful for drug discovery and basic research, and targets specific cellular compartments that may store the most disease-relevant biomarker information. This new technology overcomes the drawbacks of empirical measurements (30). Proof-of-principle examination, however, still requires independent future validation of biomarker levels.

Our study provides the first proteomics-based and functional signature of the different ARSACS cell models. Neurons are particularly sensitive to changes that disturb mitochondria dynamics, and defects in the mitochondrial compartment have been described in ARSACS (12), pointing to pathophysiological features shared with other neurodegenerative conditions (31–33). To mimic mitochondrial impairment *in vitro* in our SH-SY5Y models, we used a short-term FCCP treatment, which represents the most common method to induce full depolarization of the mitochondrial membrane and damage to mitochondrial dynamics.

Although only a single DEP was found to be common to the two cell models and its significance in ARSACS remain unclear, we showed an overlap of more than 50% of the DEPs and significant agreement with data generated in an earlier quantitative transcriptome analysis (11), further validating those findings.

In our experimental setting, we found the involvement of neuronal and neuroinflammatory processes functionally linked to target proteins, which bring us to hypothesize impaired cell functions and dysregulated expression of synapse-related genes. It is important to state that the bioinformatics categorization of dysfunctional proteins related to neuronal injury will require further studies in a disease-specific cell type (e.g., Purkinje cells) or *in vivo* (KO mice) to substantiate the specific biological function/pathway impairment. Affected pathways and related functions of *Synaptogenesis* and *Neuronal Cell Death* related to sacsin loss-of-function are known in mice, and they indicate impairment of Purkinje cells and axonal development (12, 14). It is interesting that several ephrin proteins, including EFNB2, EPHA3 and EPHB2 (28), and SNCA, the major hallmark of all synucleinopathies, accumulate in Purkinje cells in different neurodegenerative diseases such as Parkinson's disease (PD), dementia with Lewy bodies (DLB), and Alzheimer's disease (AD) (34–37), suggesting that they are critical for synaptic homeostasis in ARSACS.

*Engulfment of Cells* was also shown to be impaired in our models. Based on recent data showing that engulfment of dendrites by microglia precedes Purkinje cell degeneration in a mouse model of Niemann Pick Type-C disease (38), we are tempted to hypothesize that dysregulation of DEPs in this functional network plays a significant role in the processes of neurodegeneration in ARSACS. As an example, downregulation of ICAM5 in ARSACS might reduce neuroprotection (39), whereas low levels of SPHK1 and STUB1 could contribute to autophagy dysregulation in neurons (40, 41). The close link between functional networks defined with the aptamer-based proteomic technology, together with the observation that several DEPs also appear to be potential determinants in a large collection of unsolved patients with neurogenetic conditions, further corroborates earlier impressions that sacsin is among the hubs regulating ataxia-causing protein–protein interactions.

In SH-SY5Y model, we detected STUB1 downregulation. It is tempting to hypothesize that this finding could, at least in part, recapitulate the pathological phenotype with the impairment of Purkinje cell's homeostasis (42). Moreover, our bioinformatics analysis showed that the set of DEPs involved in the processes of *Synaptogenesis* and *Engulfment of Cells* detected in patients and KO cells are highly interconnected. Interestingly, some of these DEPs, including SNCA, APOE, and SRC were also common with the two processes. Their *bona fide* deleterious variants might play a role as modifier factors in the onset of the disease.

This study suggests that the omics approach reveals the role of modifier factors directly involved in the pathogenesis of ARSACS, pointing to putative multifactorial features of the disease, and suggesting that massive employment of omics data might overcome the traditional “one gene–one disease” paradigm.

The repurposing of FDA-approved drugs for the modulation of *Synaptogenesis, Engulfment of Cells* and *Neuronal Cell Death* could offer new options in the treatment of ARSACS, as already shown earlier by us (43). We queried bioinformatic tools to identify available druggable targets in our dataset (see Supplementary Table 4) and obtained a shortlist mainly represented by anticancer compounds. Although the connection of these compounds with neuroprotection is slim, recent studies proposed the use of some of those in brain disorders [i.e., Imatinib in multiple sclerosis (44)].

In conclusion, our novel omics strategy may have sown a seed for greater understanding of the functional network impairment underlying ARSACS and of the pathway dysregulation responsible for cell firing and Purkinje cell degeneration. The proteomic approach utilizing *in-vitro* models for a quantitative mass spectrometry is particularly appropriate to reveal specific cellular changes and affected functional modules exclusively linked to a different gene dosage and out of a physiological disease context. Certainly, our data might promote future research that will require perhaps multiomic approaches in animal models at different disease stages for a full answer on pathological processes related to the disease progression and also might address the true druggability of transspecies targets through validation in KO mice. Finally, discovery of a common dysfunctional network in untargeted studies may allow the definition of more appropriate molecular targets that may, in turn, lead to improved treatments for patients.

## Data Availability Statement

The original contributions generated for this study are publicly available. This data can be found in the Gene Expression Omnibus (GEO) under accession number GSE162132'.

## Ethics Statement

The studies involving human participants were reviewed and approved by Tuscany Pediatric Ethic Committee. The patients/participants provided their written informed consent to participate in this study.

## Author Contributions

FM, SD, GC, DG, and ES contributed to technical data. FM, SD, and FS set experiments and interpretation of results. FF, ES, EB, and SZ provided intellectual contribution. FM, FS, and FG supervised the study. FM and FS provided funds. All authors contributed to the article and approved the submitted version.

## Conflict of Interest

The authors declare that the research was conducted in the absence of any commercial or financial relationships that could be construed as a potential conflict of interest.

## References

[1] Engert JC Bérubé P Mercier J Doré C Lepage P Ge B. ARSACS , a spastic ataxia common in northeastern Québec , is caused by mutations in a new gene encoding an 11 . 5-kb ORF. Nat Genet. (2000) 24:120–5. 10.1038/72769 10655055

[2] Takiyama Y. Autosomal recessive spastic ataxia of Charlevoix-Saguenay. Neuropathology. (2006) 26:368–75. 10.1111/j.1440-1789.2006.00664.x 16961075

[3] Vermeer S Meijer RPP Pijl BJ Timmermans J Cruysberg JRM Bos MM. ARSACS in the Dutch population: a frequent cause of early-onset cerebellar ataxia. Neurogenetics. (2008) 9:207–14. 10.1007/s10048-008-0131-7 18465152 PMC2441586

[4] Synofzik M Soehn AS Gburek-Augustat J Schicks J Karle KN Schüle R. Autosomal recessive spastic ataxia of Charlevoix Saguenay (ARSACS): expanding the genetic, clinical and imaging spectrum. Orphanet J Rare Dis. (2013) 8:41. 10.1186/1750-1172-8-41 23497566 PMC3610264

[5] Baets J Deconinck T Smets K Goossens D Van den Bergh P Dahan K. Mutations in SACS cause atypical and late-onset forms of ARSACS. Neurology. (2010) 75:1181–8. 10.1212/WNL.0b013e3181f4d86c 20876471

[6] Mayne J Ning Z Zhang X Starr AE Chen R Deeke S. Bottom-up proteomics (2013-2015): keeping up in the era of systems biology. Anal Chem. (2016) 88:95–121. 10.1021/acs.analchem.5b04230 26558748

[7] Gold L Ayers D Bertino J Bock C Bock A Brody EN. Aptamer-based multiplexed proteomic technology for biomarker discovery. PLoS ONE. (2010) 5:e15004. 10.1038/npre.2010.4538.1 21165148 PMC3000457

[8] Gold L Walker JJ Wilcox SK Williams S. Advances in human proteomics at high scale with the SOMAscan proteomics platform. N Biotechnol. (2012) 29:543–9. 10.1016/j.nbt.2011.11.016 22155539

[9] Barabási A-L Gulbahce N Loscalzo J. Network medicine: a network-based approach to human disease. Nat Rev Genet. (2011) 12:56–68. 10.1038/nrg2918 21164525 PMC3140052

[10] Lim J Hao T Shaw C Patel AJ Szabó G Rual J-F. A protein-protein interaction network for human inherited ataxias and disorders of Purkinje cell degeneration. Cell. (2006) 125:801–14. 10.1016/j.cell.2006.03.032 16713569

[11] Morani F Doccini S Sirica R Paterno M Pezzini F Ricca I. Functional transcriptome analysis in ARSACS KO cell model reveals a role of sacsin in autophagy. Sci Rep. (2019) 9:11878. 10.1038/s41598-019-48047-x 31417125 PMC6695435

[12] Girard M Larivière R Parfitt DA Deane EC Gaudet R Nossova N. Mitochondrial dysfunction and Purkinje cell loss in autosomal recessive spastic ataxia of Charlevoix-Saguenay (ARSACS). Proc Natl Acad Sci U S A. (2012) 109:1661–6. 10.1073/pnas.1113166109 22307627 PMC3277168

[13] Larivière R Gaudet R Gentil BJ Girard M Conte TC Minotti S. Sacs knockout mice present pathophysiological defects underlying autosomal recessive spastic ataxia of Charlevoix-Saguenay. Hum Mol Genet. (2015) 24:727–39. 10.1093/hmg/ddu491 25260547 PMC4291249

[14] Ady V Toscano-Márquez B Nath M Chang PK Hui J Cook A. Altered synaptic and firing properties of cerebellar Purkinje cells in a mouse model of ARSACS. J Physiol. (2018) 596:4253–67. 10.1113/JP275902 29928778 PMC6117548

[15] Wilson R. High-content aptamer-based proteomics. J Proteomics. (2011) 74:1852–4. 10.1016/j.jprot.2011.04.017 21980599

[16] Rohloff JC Gelinas AD Jarvis TC Ochsner UA Schneider DJ Gold L. Nucleic acid ligands with protein-like side chains: modified aptamers and their use as diagnostic and therapeutic agents. Mol Ther - Nucleic Acids. (2014) 3:e201. 10.1038/mtna.2014.49 25291143 PMC4217074

[17] Tusher VG Tibshirani R Chu G. Significance analysis of microarrays applied to the ionizing radiation response. Proc Natl Acad Sci U S A. (2001) 98:5116–21. 10.1073/pnas.091062498 11309499 PMC33173

[18] Krämer A Green J Pollard J Tugendreich S. Causal analysis approaches in Ingenuity Pathway Analysis. Bioinformatics. (2014) 30:523–30. 10.1093/bioinformatics/btt703 24336805 PMC3928520

[19] Gonzalez M Falk MJ Gai X Postrel R Schüle R Zuchner S. Innovative genomic collaboration using the GENESIS (GEM.app) Platform. Hum Mutat. (2015) 36:950–6. 10.1002/humu.22836 26173844 PMC4682547

[20] Lieto M Riso V Galatolo D De Michele G Rossi S Barghigiani M. The complex phenotype of spinocerebellar ataxia type 48 in eight unrelated Italian families. Eur J Neurol. (2020) 27:498–505. 10.1111/ene.14094 31571321

[21] Kircher M Witten DM Jain P O'Roak BJ Cooper GM Shendure J. A general framework for estimating the relative pathogenicity of human genetic variants. Nat Genet. (2014) 46:310–5. 10.1038/ng.2892 24487276 PMC3992975

[22] Jian X Boerwinkle E Liu X. In silico prediction of splice-altering single nucleotide variants in the human genome. Nucleic Acids Res. (2014) 42:13534–44. 10.1093/nar/gku1206 25416802 PMC4267638

[23] Gruol DL Nelson TE. Purkinje neuron physiology is altered by the inflammatory factor interleukin-6. Cerebellum. (2005) 4:198–205. 10.1080/14734220500199987 16147952

[24] Limatola C Giovannelli A Maggi L Ragozzino D Castellani L Ciotti MT. SDF-1alpha-mediated modulation of synaptic transmission in rat cerebellum. Eur J Neurosci. (2000) 12:2497–504. 10.1046/j.1460-9568.2000.00139.x 10947825

[25] Lazarini F Tham TN Casanova P Arenzana-Seisdedos F Dubois-Dalcq M. Role of the alpha-chemokine stromal cell-derived factor (SDF-1) in the developing and mature central nervous system. Glia. (2003) 42:139–48. 10.1002/glia.10139 12655598

[26] Solmaz V Köse Özlece H Eroglu HA Aktug H Erbaş O Taşkiran D. Accumulation of α -synuclein in cerebellar purkinje cells of diabetic rats and its potential relationship with inflammation and oxidative stress markers. Neurol Res Int. (2017) 2017:1–6. 10.1155/2017/5952149 28133547 PMC5241473

[27] Lee H Cho S-J Moon IS. The non-canonical effect of N-acetyl-D-glucosamine kinase on the formation of neuronal dendrites. Mol Cells. (2014) 37:248–56. 10.14348/molcells.2014.2354 24625575 PMC3969046

[28] Klein R Kania A. Ephrin signalling in the developing nervous system. Curr Opin Neurobiol. (2014) 27:16–24. 10.1016/j.conb.2014.02.006 24608162

[29] Ricca I Morani F Bacci GM Nesti C Caputo R Tessa A. Clinical and molecular studies in two new cases of ARSACS. Neurogenetics. (2019) 20:45–9. 10.1007/s10048-019-00564-7 30680480

[30] Joshi A Mayr M. In aptamers they trust. Circulation. (2018) 138:2482–5. 10.1161/CIRCULATIONAHA.118.036823 30524136 PMC6277005

[31] Chen H Chan DC. Mitochondrial dynamics-fusion, fission, movement, and mitophagy-in neurodegenerative diseases. Hum Mol Genet. (2009) 18:R169–76. 10.1093/hmg/ddp326 19808793 PMC2758711

[32] Schon EA Przedborski S. Mitochondria: the next (neurode)generation. Neuron. (2011) 70:1033–53. 10.1016/j.neuron.2011.06.003 21689593 PMC3407575

[33] Wang X Su B Lee H Li X Perry G Smith MA. Impaired balance of mitochondrial fission and fusion in Alzheimer's disease. J Neurosci. (2009) 29:9090–103. 10.1523/JNEUROSCI.1357-09.2009 19605646 PMC2735241

[34] Mori F Piao Y-S Hayashi S Fujiwara H Hasegawa M Yoshimoto M. α-Synuclein accumulates in purkinje cells in lewy body disease but not in multiple system atrophy. J Neuropathol Exp Neurol. (2003) 62:812–9. 10.1093/jnen/62.8.812 14503637

[35] Sebeo J Hof PR Perl DP. Occurrence of alpha-synuclein pathology in the cerebellum of Guamanian patients with parkinsonism-dementia complex. Acta Neuropathol. (2004) 107:497–503. 10.1007/s00401-004-0840-4 15024581

[36] Nussbaum RL. Genetics of synucleinopathies. Cold Spring Harb Perspect Med. (2018) 8:a024109. 10.1101/cshperspect.a024109 28213435 PMC5983162

[37] Khan SS LaCroix M Boyle G Sherman MA Brown JL Amar F. Bidirectional modulation of Alzheimer phenotype by alpha-synuclein in mice and primary neurons. Acta Neuropathol. (2018) 136:589–605. 10.1007/s00401-018-1886-z 29995210 PMC6329667

[38] Kavetsky L Green KK Boyle BR Yousufzai FAK Padron ZM Melli SE. Increased interactions and engulfment of dendrites by microglia precede Purkinje cell degeneration in a mouse model of Niemann Pick Type-C. Sci Rep. (2019) 9:14722. 10.1038/s41598-019-51246-1 31605022 PMC6788982

[39] Birkner K Loos J Gollan R Steffen F Wasser B Ruck T. Neuronal ICAM-5 plays a neuroprotective role in progressive neurodegeneration. Front Neurol. (2019) 10:205. 10.3389/fneur.2019.00205 30915022 PMC6422935

[40] Moruno Manchon JF Uzor N-E Finkbeiner S Tsvetkov AS. SPHK1/sphingosine kinase 1-mediated autophagy differs between neurons and SH-SY5Y neuroblastoma cells. Autophagy. (2016) 12:1418–24. 10.1080/15548627.2016.1183082 27467777 PMC4968226

[41] Sha Y Rao L Settembre C Ballabio A Eissa NT. STUB1 regulates TFEB–induced autophagy–lysosome pathway. EMBO J. (2017) 36:2544–52. 10.15252/embj.201796699 28754656 PMC5579343

[42] Shi Y Wang J Li J-D Ren H Guan W He M. Identification of CHIP as a novel causative gene for autosomal recessive cerebellar ataxia. PLoS ONE. (2013) 8:e81884. 10.1371/journal.pone.0081884 24312598 PMC3846781

[43] Ricca I Tessa A Trovato R Bacci GM Santorelli FM. Docosahexaenoic acid in ARSACS: observations in two patients. BMC Neurol. (2020) 20:215. 10.1186/s12883-020-01803-3 32466761 PMC7254735

[44] Rotstein DL Sawicka K Bharatha A Montalban X Lipton JH. CNS demyelination after initiating the tyrosine kinase inhibitor imatinib: a report of two cases. Mult Scler. (2020) 26:1121–4. 10.1177/1352458519892914 31845621

