# Supplementary material for: Functional Network Profiles in ARSACS Disclosed by Aptamer-Based Proteomic Technology
Source: Front Neurol. 2021 Jan 27;11:603774. doi: 10.3389/fneur.2020.603774 (PMC7873355; doi:10.3389/fneur.2020.603774)
Supplement: Supplementary file 1 [file Table_1.DOC]

| **SAMPLES** | **Origin** | **Genotype** | **Phenotype** | **Protein expression** |
| --- | --- | --- | --- | --- |
| Pt 1 | Skin fibroblasts from an ARSACS patient | Homozygous deletion  c.2187-4345_9832del | Severe | none |
| Pt 2 | Skin fibroblasts from an ARSACS patient | c.4568G>A (p.W1523*);  c.9305T>A (p.L3102*) | Severe | none |
| Pt 3 | Skin fibroblasts from an ARSACS patient | c.2343insT;  c.5017C>T (p.R895*) | Severe | none |

**Supplementary Table S1.** Characterization of ARSACS samples.
